# Dentinogenic Ghost Cell Tumor of the Peripheral Variant Mimicking Epulis

**DOI:** 10.1155/2010/519494

**Published:** 2010-10-26

**Authors:** Uddipan Kumar, Hitesh Vij, Ruchieka Vij, Jitin Kharbanda, IN Aparna, Raghu Radhakrishnan

**Affiliations:** ^1^Department of Oral Pathology and Microbiology, Hazaribag College of Dental Sciences and Hospital, Hazaribag 825 301, Jharkhand, India; ^2^Department of Oral Pathology, Institute of Dental Studies and Technologies, Modinagar, Uttar Pradesh 201 201, India; ^3^Department of Oral Pathology, Shree Bankey Bihari Dental College, Masuri, Ghaziabad 201 302, India; ^4^Department of Prosthodontics, Manipal College of Dental Sciences, Manipal University, Manipal 576 104, India; ^5^Department of Oral Pathology and Microbiology, Manipal College of Dental Sciences, Manipal University, Manipal 576 104, India

## Abstract

Dentinogenic ghost cell tumor (DGCT) is an uncommon locally invasive odontogenic tumor regarded by many as a variant of calcifying odontogenic cyst. The peripheral variant of this clinical rarity appears as a well-circumscribed mass mimicking a nonspecific gingival enlargement. Microscopic appearance of odontogenic epithelium admixed with focal areas of dentinoid formation and sheets of ghost cells giving the definitive diagnosis of dentinogenic ghost cell tumor imply that microscopic examination is compulsory for any gingival mass. Van Gieson histochemical stain further confirmed the nature of dentinoid-like material. A complete workup of a case of peripheral dentinogenic ghost cell tumor is presented in this paper and the current concept as well as the appraisal of literature is presented.

## 1. Introduction

Calcifying odontogenic cyst (COC) is a unique jaw lesion, first recognized as a distinct entity by Gorlin et al. [1] and hence the eponym Gorlin cyst. Praetorius et al. [2] classified them into the cystic type (Type I) and the solid type (Type II). The solid variant of COC (Type II) is rare and is designated as dentinogenic ghost cell tumor (DGCT), although the first description of the solid variant was given by Fejerskov and Krogh as calcifying ghost cell odontogenic tumor [3]. DGCT is characterized microscopically by ameloblastoma-like odontogenic epithelial proliferation, presence of ghost cells, and dentinoid-like material [4]. Due to its diverse histological picture, several terms have been used by different authors to describe this lesion such as dentinogenic ghost cell tumor [2], calcifying ghost cell odontogenic tumor [3], keratinizing ameloblastoma [5], cystic calcifying odontogenic tumor [6], peripheral odontogenic tumor with ghost cell keratinization [7], dentinoameloblastoma [8], ameloblastic dentinoma [9], epithelial odontogenic ghost cell tumour [10], and odontogenic ghost cell tumor [11].

The term DGCT is commonly used, and the peripheral variant of this neoplastic entity is rare; only few reports with clinical, radiographic, and histologic documentation can be found in the English literature. A report of peripheral dentinogenic ghost cell tumor (PDGCT) and characterization of dentinoid material using Van Gieson special stain for the confirmation adds a new dimension to the diagnosis of DGCT.

## 2. Case Report

**Figure 1:**
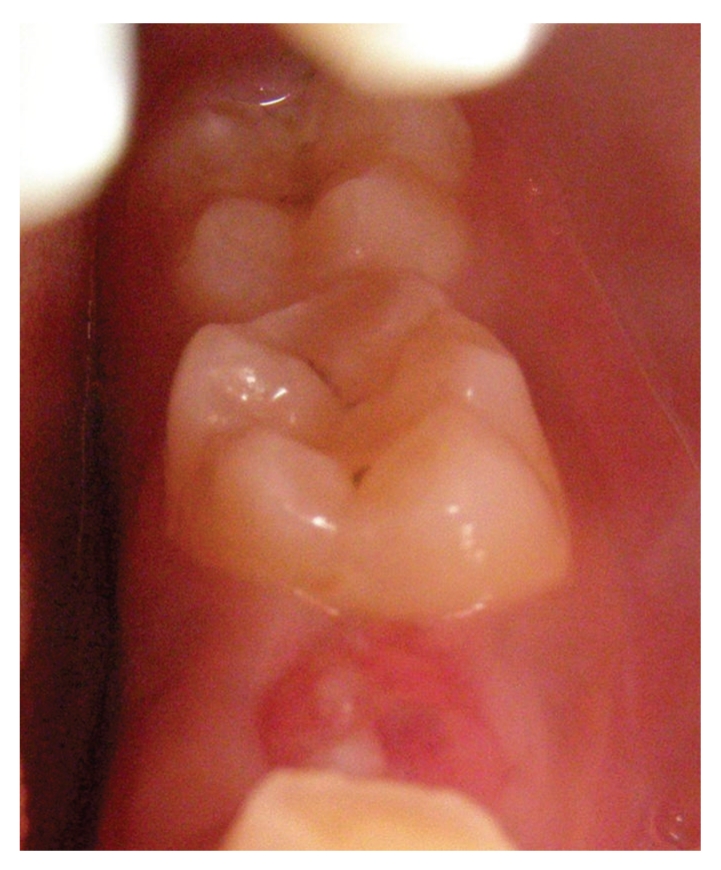
A nontender, persistent swelling in the region of lower left posterior region.

A 40-year-old male patient reported to the dental clinics at Manipal College of Dental Sciences, Manipal University, with a complaint of missing teeth. Clinical examination disclosed a swelling measuring about 5 mm in diameter in the region of lower left premolars which was non tender, and the over lying mucosa was normal in color. Clinically, a provisional diagnosis of epulis was made (Figure 1). The lesion was excised under local anesthesia, and the tissue was submitted for histopathological examination. Six months of followup examination did not show any signs of recurrence.

**Figure 2:**
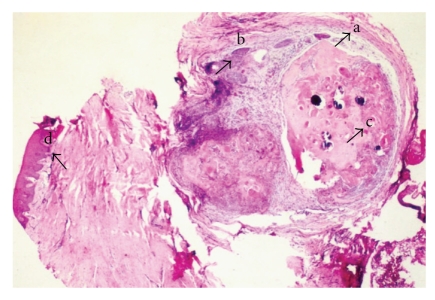
Solid wellcircumscribed encapsulated mass (a) showing ameloblastomatous islands of odontogenic epithelium (b) and ghost cells (c) with overlying epithelium (d) [Hematoxylin and Eosin, ×4].

**Figure 3:**
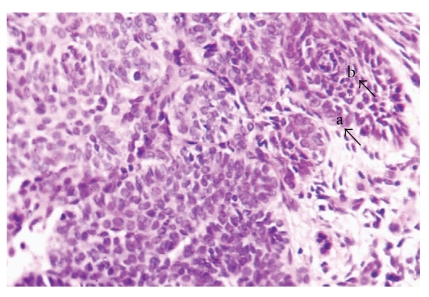
Ameloblastomatous island of odontogenic epithelium with columnar basal cells (a) enclosing stellate reticulum like cells (b) [Hemataoxylin and Eosin, ×40].

**Figure 4:**
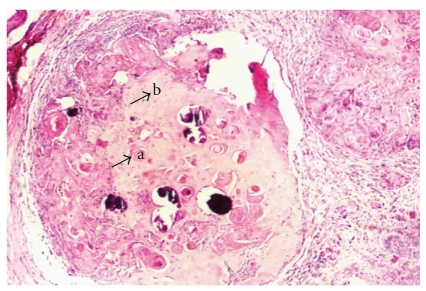
Ghost cells (a) and surrounding dentinoid-like material (b) [Hemataoxylin and Eosin, ×20].

**Figure 5:**
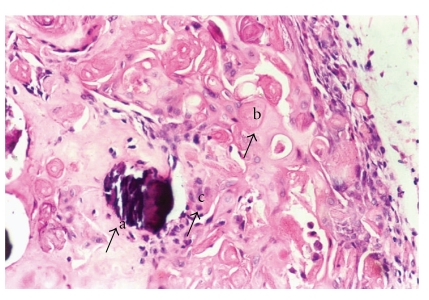
Irregular foci of dentine/osteo-dentine-like material (a) and calcifying ghost cells (b) and giant cells (c) [Hemataoxylin and Eosin, ×40].

**Figure 6:**
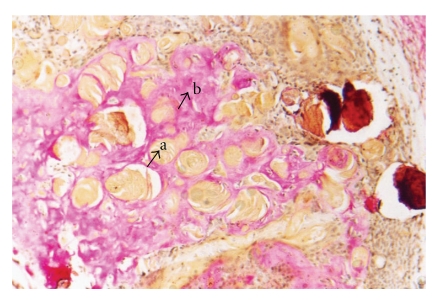
Van Gieson stains showing ghost cells staining yellow (a) and surrounding dentinoid-like material staining pink (b) [Van Gieson stain, ×40].

Histological examination revealed a solid well-circumscribed, encapsulated soft tissue mass surrounded by a dense fibrous connective tissue covered by stratified squamous epithelium (Figure 2). The tumor mass revealed islands of odontogenic epithelium resembling follicles of ameloblastoma, consisting of columnar cells enclosing stellate reticulum like cells (Figure 3). These elements were associated with numerous pale, eosinophilic ghost cells with granular eosinophilic cytoplasm and faint nuclear outline (Figure 4). Few multinucleated giant cells of foreign body type were evident at the periphery of the ghost cells in the connective tissue stroma. Irregular foci of tissue resembling dentin were observed surrounding the odontogenic epithelial islands, and these areas were atubular and at places showed cellular inclusions (Figure 5). Van Gieson special stain was carried out to examine the nature of the dentinoid-like material (Figure 6). The characteristic microscopic features and the confirmation of dentinoid-like material by special stain contributed to the diagnosis of dentinogenic ghost cell tumor of the peripheral variant.

## 3. Discussion

DGCT is a distinct but a rare histological entity among odontogenic ghost cell lesions which have been recently classified into the simple cystic type or COC; cysts associated with odontogenic hamartomas or benign neoplasms; solid benign odontogenic neoplasm, which is same as COC but with dentinoid formation, the DGCT; malignant odontogenic neoplasm-ghost cell odontogenic carcinoma [8]. The peripheral dentinogenic ghost cell tumor (PDGCT), though a distinct entity of odontogenic origin, is apparently rare. This rarity is due to the failure of its recognition as an isolated entity [12], and many of the cases of PDGCT have been mistakenly diagnosed as peripheral ameloblastoma [13].

The usual presentation of the peripheral variant is a nodular swelling on the edentulous alveolar mucosa of denture wearers, a feature that implicates trauma/irritation. This clinical presentation could lead to the provisional diagnosis of epulis as was true in our case as well. Due to the dearth of documented cases, it is difficult to typify the exact location, age, and gender distribution of PDGCT. However based on the existing literature it appears to be more common in the canine-premolar region, with a predilection for the elderly age group [2, 9, 12, 14–23] (Table 1). Hirshberg et al. [16] indicated that the tumor mainly affects the maxilla and occurs four times more commonly among men. Contrary to this, Hong et al. [24] reported that it mainly occurs in the mandibular edentulous mucosa without any sex predilection. This lesion seems to be less aggressive than its central counterpart with no recurrences reported after excision [12].

Based on this histological diversity, the origin of this lesion has been attributed to cell rests of Serres or the surface epithelium [25, 26]. The tumor is mainly composed of ameloblastoma-like areas of odontogenic epithelial islands with varying amount of ghost cells showing keratinization and calcification. It may histologically show areas similar to or may be associated with odontogenic tumors like complex and compound odontomes, ameloblastic fibro-odontoma, and so forth. Ghost cells are thought to be transformed odontogenic epithelial cells, the mechanism of whose transformation remains unknown [8]. In light microscope, they appear as enlarged, ovoid, or elliptoid epithelial cells, which are eosinophilic, usually with well-defined cell outlines but may be blurred giving them a fused appearance. Histochemically, they are positive for keratin giving a yellow fluorescence with Rhodamine B. Sometimes ghost cells may contain nuclear remnants in various stages of degeneration. The calcification which may occur in some of these ghost cells appear initially as fine powdery/coarse basophilic granules and later as small spherical bodies. Ultrastructural studies have shown that these calcifications represent dystrophic calcification [27].

Due to its biological behavior COC, and its variants are thought to resemble a benign odontogenic tumor rather than a cyst [6]. Previously, Abrams and Howell [14] have established that the term “cyst” does not imply in all instances as its clinical features, growth behavior, and recurrence potential are quite similar to those of ameloblastoma. It has also been emphasized that calcifications found in COC and CEOT are not a feature of ameloblastoma and the combined occurrence of COC with other odontogenic lesions could be due to the multipotentiality of odontogenic epithelium [4]. Further it is pointed out that this lesion in association with an odontoma may result due to differentiation and degeneration of odontogenic epithelium [14]. What appears as associated odontogenic tumor should be considered as an integral part of an entire lesion developing in the wall of COC [2]. 

In our case as well, the presence of keratinizing ghost cells and mineralization was an outstanding feature. The lesion did not exhibit any cystic areas, and its features were consistent with those of DGCT. The calcifications seen as dentoid, frequently described in connection with masses of ghost cells, were a characteristic finding of this lesion. These calcified areas were thought to represent juxtaepithelial osteoid formation by granulation tissue due to induction by ghost cells [14]. This view was however negated later as juxtaepithelial osteoid and dentinoid were observed in areas free of granulation tissue and ghost cells, as pointed out by Tajima et al. [28]. There are no reports or studies to clarify if this is an inductive effect or a metaplastic change in the connective tissue. In the present case as well, the calcified material was found either in areas where the ghost cells and connective tissue were in contact or in the connective tissue adjacent or below the basal cells, possibly due to the inductive effects of ghost cells/epithelial cells [29]. Van Gieson special stain illustrated the collagenous nature of the dentinoid-like material. The histological and histochemical attributes of the dentinoid-like calcification described in this peripheral odontogenic tumor best fitted with the diagnosis of Peripheral Dentinogenic Ghost Cell Tumor. 

Conservative but aggressive local resection has been the treatment of choice with no recurrences found in any patient. However patients with a DGCT should remain in long-term follow-up [30]. Though numerous terminologies have been suggested for this entity, we propose that for a lesion with the above histological and histochemical characteristics, Peripheral Dentinogenic Ghost Cell Tumor best describes it and should be the most preferred designation.

## Figures and Tables

**Table 1:** Reported cases of peripheral dentinogenic ghost cell tumor.

Sl no.	Reference	No. of cases	Age in years/sex	Site	Radiographic features
1	Abrams and Howell [14]	1	13/M	Extraosseous	No significant findings
2	Sauk [15]	1	67/F	Extraosseous	No radiographic changes
3	Praetorius et al. [2]	2	52/M	Maxillary left lateral incisor and canine region	Slight erosion of underlying bone
			41/F	Mandibular anterior region	Slight erosion of underlying bone
4	Hirshberg et al. [16]	1	42/M	Mandibular left premolar region (lingual gingiva)	No bone involvement
5	McClatchey et al. [17]	1	57/M	Mandibular anterior region	No bone involvement
6	Buchner et al. [12]	3	10/M	Mandibular central incisor	—
			53/F	Mandibular edentulous ridge	—
			92/F	Mandibular edentulous cuspid-premolar region	—
7	Günhan et al. [18]	1	71/F	Maxillary anterior region	Slight erosion of underlying bone
8	Raubenheimer et al. [19]	1	82/M	Mandibular right alveolar ridge (edentulous)	No bone involvement
9	Castro et al. [20]	1	83/F	Anterior ridge of edentulous mandible	Cup shaped resorption
10	Wong et al. [9]	1	71/M	Maxillary canine region	Slight erosion beneath the growth
11	Iezzi et al. [21]	1	43/M	Maxillary canine region	No bone involvement
12	Ledesma-Montes et al. [22]	1	NS	NS	NS
13	Candido et al. [23]	1	45/M	Mandibular canine region	No bone involvement
14	Our case	1	40/M	Mandibular premolar area	—

NS: Not Specified.

## Abbreviations

DGCT: Dentinogenic ghost cell tumor
COC: Calcifying odontogenic cyst
PDGCT: Peripheral dentinogenic ghost cell tumor.
